# Effect of apparent temperature on daily emergency admissions for mental and behavioral disorders in Yancheng, China: a time-series study

**DOI:** 10.1186/s12940-019-0543-x

**Published:** 2019-11-20

**Authors:** Min Min, Tingting Shi, Pengpeng Ye, Yuan Wang, Zhenhai Yao, Shun Tian, Yun Zhang, Mingming Liang, Guangbo Qu, Peng Bi, Leilei Duan, Yehuan Sun

**Affiliations:** 1 Department of Epidemiology and Health Statistics, School of Public Health, Anhui Medical University, Hefei, 230032 Anhui China; 2 Center for chronic noncommunicable diseases, Chinese center for disease control and prevention, Beijing, 100050 China; 3 Anhui public meteorological service center, Hefei, Anhui, 230011 China; 4 Preventive medicine, School of Public Health, Anhui Medical University, Hefei, 230032 Anhui China; 5 School of Public Health, University of Adelaide, Adelaide, SA 5005 Australia

**Keywords:** Apparent temperature, Hospital emergency admissions, Mental and behavioral disorders, Time-series analysis

## Abstract

**Background:**

Very few studies have focused on the relationship between ambient apparent temperature (AT) and admission of mental and behaviour disorders (MDs). Therefore, a time-series study was conducted in Yancheng, China, to explore the effects of AT on the daily emergency admissions of patients with MDs over the period of 2014–17.

**Methods:**

A quasi-Poisson generalized linear model (GLM) combined with a distributed lag non-linear model (DLNM) was adopted to explore the associations after adjusting for time trend, day of the week, humidity, sunshine duration, rainfall, holidays and air pollutants. In the subgroup analysis, the modification effects of age and sex were also examined.

**Results:**

Overall, 8438 cases of MDs emergency admissions were identified. With the apparent temperature with the minimum number of admissions (− 3.4 °C) serving as a reference, a positive correlation emerged between high AT and daily emergency admissions of patients with MDs in Yancheng, China, with the lagged effect of 1 to 5 days. The subgroup analysis demonstrated a positive relationship between AT and MDs emergency admissions among males and individuals younger than 45 years old, with no lagged effect.

**Conclusions:**

The results will provide important scientific evidence for mental health policy-makers and practitioners for possible intervention, especially among the vulnerable populations.

## Background

Mental disorders (MDs) include depression, bipolar disorder, schizophrenia, mental disability and developmental disorders, including autism [1], and collectively account for approximately 6.2% of the total global disease burden when measured in disability-adjusted life years (DALYs) [2]. Worldwide, approximately 350 million people suffer from depression, 60 million people from bipolar affective disorder, 47.5 million from dementia, and 21 million from schizophrenia and other psychoses [1]. In China, the lifetime prevalence of all mental disorders except for dementia is approximately 16.6% [3], with total costs of $3665 for each individual patient and $88.8 billion for society as a whole in 2013 [4]. Previous studies have indicated that exposures to ambient particulate matter (PM_10_; PM_2.5_), nitrogen dioxide (NO_2_) and sulphur dioxide (SO_2_) are considered risk factors for MDs [5–7]. Ambient temperature is frequently reported to be associated with mental health around the world [8–11]. Apparent temperature (AT) is a composite indicator of ambient temperature, relative humidity and wind velocity, reflecting human thermal perception more objectively than temperature itself [12]. In addition, it has been reported to be more closely associated with mortality than other temperature variables [13]. While most of the above studies use temperature and mental health hospitalization as study variables, there have been a limited number of studies using AT to examine its effects on MDs emergency admissions, especially in developing countries, including China. Therefore, we conducted a time series study to explore the lag-exposure-response relationship between AT and the risk of emergency admissions of patients with MDs in Yancheng, China, to provide scientific evidence for mental health prevention and intervention.

## Method

### Study area and data collection

The study area was located in Yancheng, a city of approximately 8 million people. Yancheng lies in the middle of the eastern coast of China (33°38′E, 120°13′N) and has a typical subtropical monsoon climate with four distinct seasons.

Daily records of emergency admissions for MDs (ICD-10 codes: F00–F99) from January 1, 2014, to December 31, 2017, were obtained from the hospital medical record systems of Yancheng city and included the date of admission and patients’ age, gender, and occupation.

Daily meteorological data were collected from the China Meteorological Administration from 2014 to 2017, including daily average temperature, daily maximum temperature, daily minimum temperature, relative humidity, wind velocity, rainfall, sunshine duration and barometric pressure. Daily air pollution data for Yancheng, including PM_2.5_, PM_10_, SO_2_, NO_2_, CO and O_3_, were obtained from the website of the Environmental Monitoring Center. The apparent temperature was calculated with common meteorological indicators (average temperature, relative humidity and wind velocity) using the following specific formulas [14]:

1 $$ \mathrm{AT}=\mathrm{T}+0.33\ast \mathrm{e}-0.70\ast \mathrm{WS}-4.00 $$

2 $$ \mathrm{e}=\mathrm{Rh}/100\ast 6.105\ast \exp .\left(17.27\ast \mathrm{T}/\left(237.7+\mathrm{T}\right)\right) $$

In the above formulas, T denotes the ambient average temperature (°C); e refers to water vapor pressure (hPa), which was calculated with the ambient average temperature and relative humidity using eq. (2); WS denotes wind speed (m/s); and Rh is relative humidity (%).

Prior to data collection, this study was approved by the ethics committee of the Chinese Centers for Disease Control and Prevention Institute for Environmental Health and Related Product Safety (201606).

### Statistical analysis

A generalized liner model (GLM) following a quasi-Poisson distribution was applied, considering the over-dispersion counts of daily emergency admissions of patients with MDs, and the log function was used as the link function [15]. Spearman’s correlation coefficients < 0.7 were used to select covariates to avoid multicollinearity. The Spearman correlations of different meteorological factors and air pollution are shown in Additional file 1: Figure S1. Finally, barometric pressure and PM_10_ were excluded, and daily mean temperature, relative humidity, sunshine duration, rainfall, PM_2.5_, SO_2_, NO_2_, and O_3_ were included. Moreover, the variance inflation factors (VIFs) for these variables were 2.108, 2.304, 1.905, 1.174, 2.464, 2.570, 2.003 and 1.777, respectively.

$$ \mathrm{Yt}\sim \mathrm{quasi}\ \mathrm{Poison}\ \left(\upmu \mathrm{t}\right) $$

$$ {\displaystyle \begin{array}{l}\mathrm{Log}\ \left(\upmu \mathrm{t}\right)=\upalpha +\upbeta\ \mathrm{ATt},1+\mathrm{ns}\ \left(\mathrm{Time},\mathrm{df}=4\ast 4\right)+\mathrm{ns}\ \left(\mathrm{SDt},\mathrm{df}=3\right)+\mathrm{ns}\ \left(\mathrm{Rht},\mathrm{df}=3\right)+\mathrm{ns}\\ {}\left(\mathrm{Rainfallt},\mathrm{df}=3\right)+\upeta \mathrm{DOWNt}+\upgamma \mathrm{Holidayt}+\mathrm{ns}\ \left({\mathrm{PM}}_{2.5},\mathrm{df}=5\right)\end{array}} $$

where Yt is the expected count of MDs cases for day t; α is the model intercept; AT_t,l_ is the DLNM cross-basis matrix of apparent temperature; l is the number of lag days; β is the vector of regression coefficients for AT_t,l_; and ns() is the natural cubic spline. Additionally, 4 degrees of freedom (df) per year is used for time; 3 df is used to adjust for possible impacts from humidity, sunshine duration, rainfall and air pollutants. DOW is the day of the week, with a reference day of Friday. Public holidays are also accounted for through the use of the categorical variable holiday.

According to the combination of AIC criteria and relevant literature, we chose 21 days as the maximum lag period in the model. The AIC values of lag1 to lag30 days are listed in Table 1. In the case of a nonlinear relationship [16], we calculated the relative risk (RR) with a 95% confidence interval (CI) of specific ATs (10th percentile, 90th percentile) on daily MDs emergency admissions, with the AT corresponding to the minimum number of admissions as the reference (i.e., the AT with the lowest risk of total MDs admissions). Further analysis was conducted through stratification by sex and age group. Sensitivity analysis was performed by changing the df for time (4–6 df/year), sunshine duration (3–5 df), humidity (3–5 df), rainfall (3–5 df), and air pollutants (3–5 df). All statistical analyses were performed using R software (Version 3.5.3) and the “dlnm” and “splines” packages [15].

**Table 1:** The AIC values of models for various lag periods from lag1 to lag 30 days

Lag (days)	1	2	3	4	5	6
AIC	7135.102	7125.197	7123.229	7111.478	7105.905	7109.386
Lag (days)	7	8	9	10	11	12
AIC	7106.372	7102.363	7099.484	7098.49	7092.935	7087.281
Lag (days)	13	14	15	16	17	18
AIC	7072.434	7069.653	7066.486	7061.328	7058.34	7054.769
Lag (days)	19	20	21	22	23	24
AIC	7048.753	7040.924	7037.055	7036.359	7025.311	7017.717
Lag (days)	25	26	27	28	29	30
AIC	7012.755	7007.583	6997.914	6991.915	6989.067	6985.077

## Results

In total, 8438 hospital MDs emergency admissions were included over the study period, which encompassed 6802 admissions of patients with MDs due to alcohol use. Males and patients younger than 45 years accounted for 69.8 and 71.6%, respectively, of MDs emergency admissions. Patients aged 45–60 years and older than 60 years accounted for 22.6 and 5.8%, respectively. There were more MDs emergency admissions in cool seasons than in warm seasons (4304 vs. 4134). The daily AT and mean temperatures in Yancheng over the study period were 15.2 °C and 15.7 °C, respectively. Characteristics of meteorological variables, pollutants and cases are shown in Table 2. The time series distributions of the total MDs cases, mean temperatures and ATs from 2014 to 2017 are presented in Additional file 1: Fig. S2, indicating a slight seasonal pattern.

**Table 2:** Characteristics of admissions for MDs and meteorological variables and air pollutants in Yancheng, China, 2014–17

Group	Sum	Mean (SD)	P1	P5	P10	P25	P50	P75	P90	P95	P99
Total (F00–F99)	8438	5.8 (3.0)	0	2	2	4	5	8	10	11	14.4
MDs due to alcohol (F10)	6802	4.7 (2.7)	0	0	1	2	4	6	8	10	13
Male	5889	4.1 (2.4)	0	1	1	2	4	5	7	8	11
Female	2549	1.7 (1.5)	0	0	0	1	2	2	4	4	6
< 45 years	6038	4.1 (2.4)	0	1	1	2	4	6	7	9	11
≥ 45 years	2400	1.6 (1.4)	0	0	0	1	1	2	3	4	6
45–60 years	1911	1.3 (1.2)	0	0	0	0	1	2	3	4	5
≥ 60 years	489	0.3 (0.6)	0	0	0	0	0	1	1	1	2
Warm (Apr to Sep)	4134	2.8 (3.5)	0	0	0	0	0	5	8	9	12
Cool (Oct to Mar)	4304	2.9 (3.7)	0	0	0	0	0	5	8	10	14
Apparent temperature (°C)	–	15.2 (12.1)	−5.4	−2.8	−0.92	4.03	15.6	25.3	30.6	34.5	38.1
Mean temperature (°C)	–	15.7 (9.1)	−0.7	1.3	3.1	7.3	16.7	23.4	27.0	29.3	32.2
Relative humidity (%)	–	76.1 (13.4)	45	51	56	67	78	86	93	96	100
Sunshine duration (h)	–	5.2 (3.9)	0	0	0	0	6.0	8.5	10.1	10.9	11.8
Wind speed (m/s)	–	2.5 (1.1)	0.6	1.1	1.3	1.7	2.3	3.0	3.8	4.3	5.6
Rainfall (mm)	–	3.3 (12.5)	0	0	0	0	0.0	0.3	8.1	20.2	60.7
BP (hPa)	–	1017.0 (9.2)	999.9	1003	1005	1009	1017	1024	1029	1032	1035
PM_10_ (μg/m^3^)	–	83.3 (49.0)	15.6	28.0	34.0	48.0	71.0	108.0	146.0	176.0	253.0
PM_2.5_ (μg/m^3^)	–	48.3 (35.0)	8.0	12.0	15.0	230.0	23.0	39.0	94.0	115.0	178.4
SO_2_ (μg/m^3^)	–	16.9 (10.1)	5	7	8	10	14	20	30	37	54
CO (mg/m^3^)	–	0.8 (0.3)	0.3	0.4	0.5	0.6	0.7	0.9	1.2	1.4	1.8
NO_2_ (μg/m^3^)	–	25.7 (13.1)	8	11	12	16.0	22.0	31.0	44	54	67
O_3__8h (μg/m^3^)	–	105.8 (38.0)	29.6	56.0	64.0	78.0	101.0	127.0	156.0	175.0	212.4

P1, P5, P10, P25, P50, P75, P90, P95, P99: the 1th percentile, the 5th percentile, the 10th percentile, the 25th percentile, the 50th percentile, the 75th percentile, the 90th percentile, the 95th percentile, the 99th percentile; SD, standard deviation; BP, barometric pressure

**Fig. 1:**
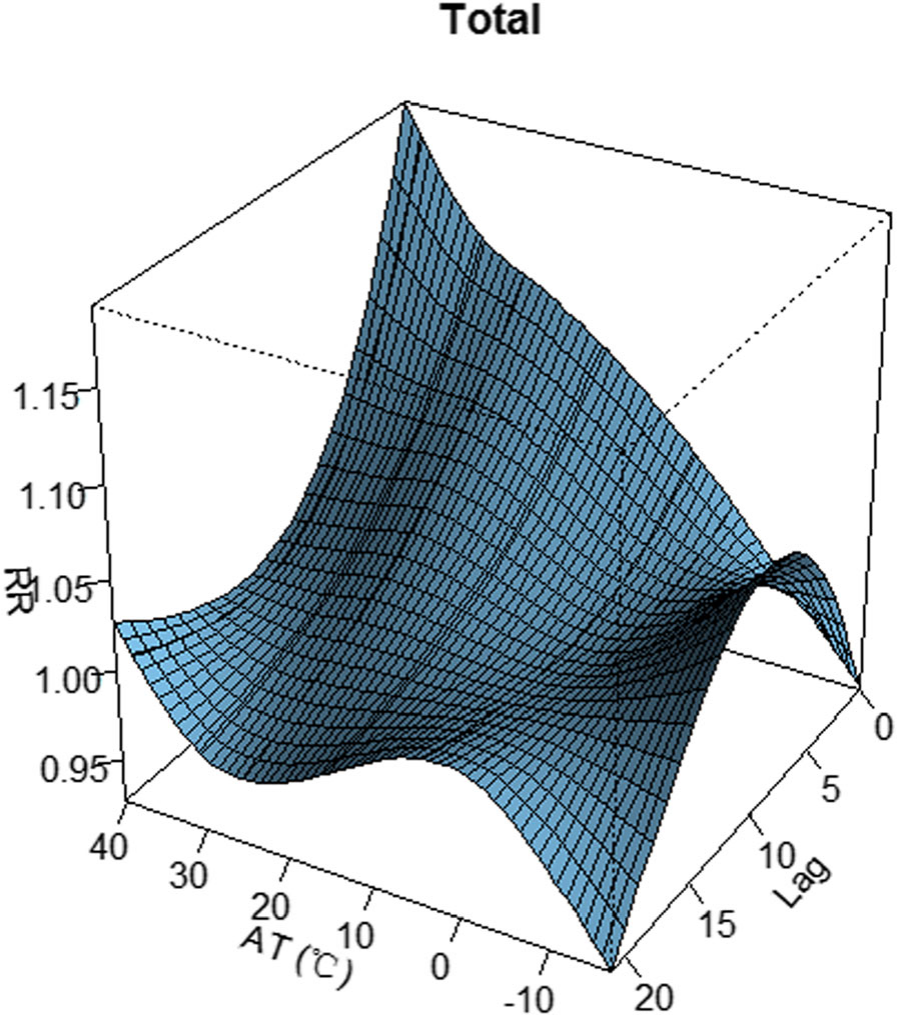
Three-dimension plot for relative risk (RR) of MDs along apparent temperature (AT) and lags produced by DLNM in Yancheng, China, 2014–17

**Fig. 2:**
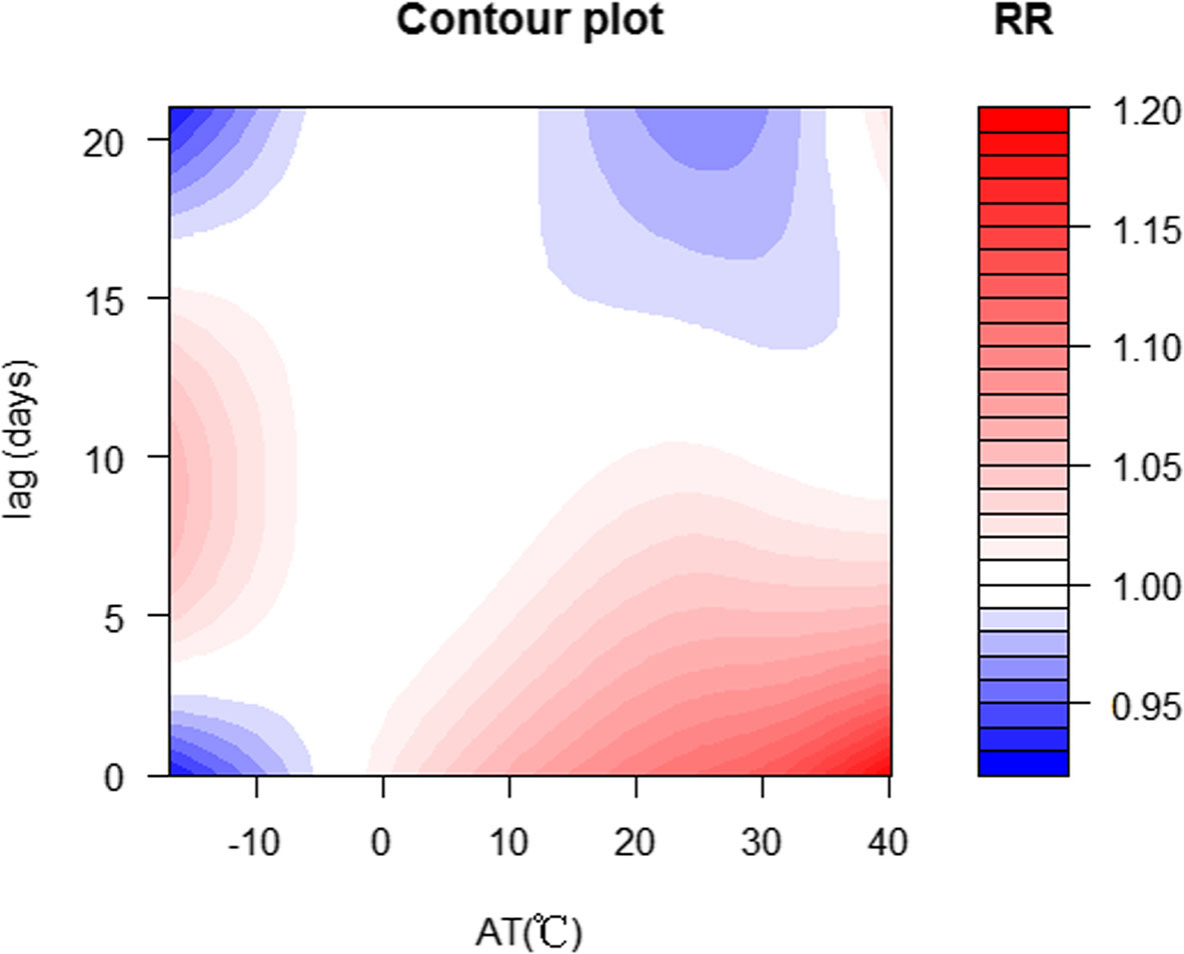
Heat map for relative risk (RR) of MDs along AT and lags produced by DLNM in Yancheng, China, 2014–17

The exposure-response association between MDs admissions and daily AT and lag days is shown in Figs. 1 and 2, qualitatively indicating that high AT had acute effects and lagged effects on admissions of patients with MDs.

**Fig. 3:**
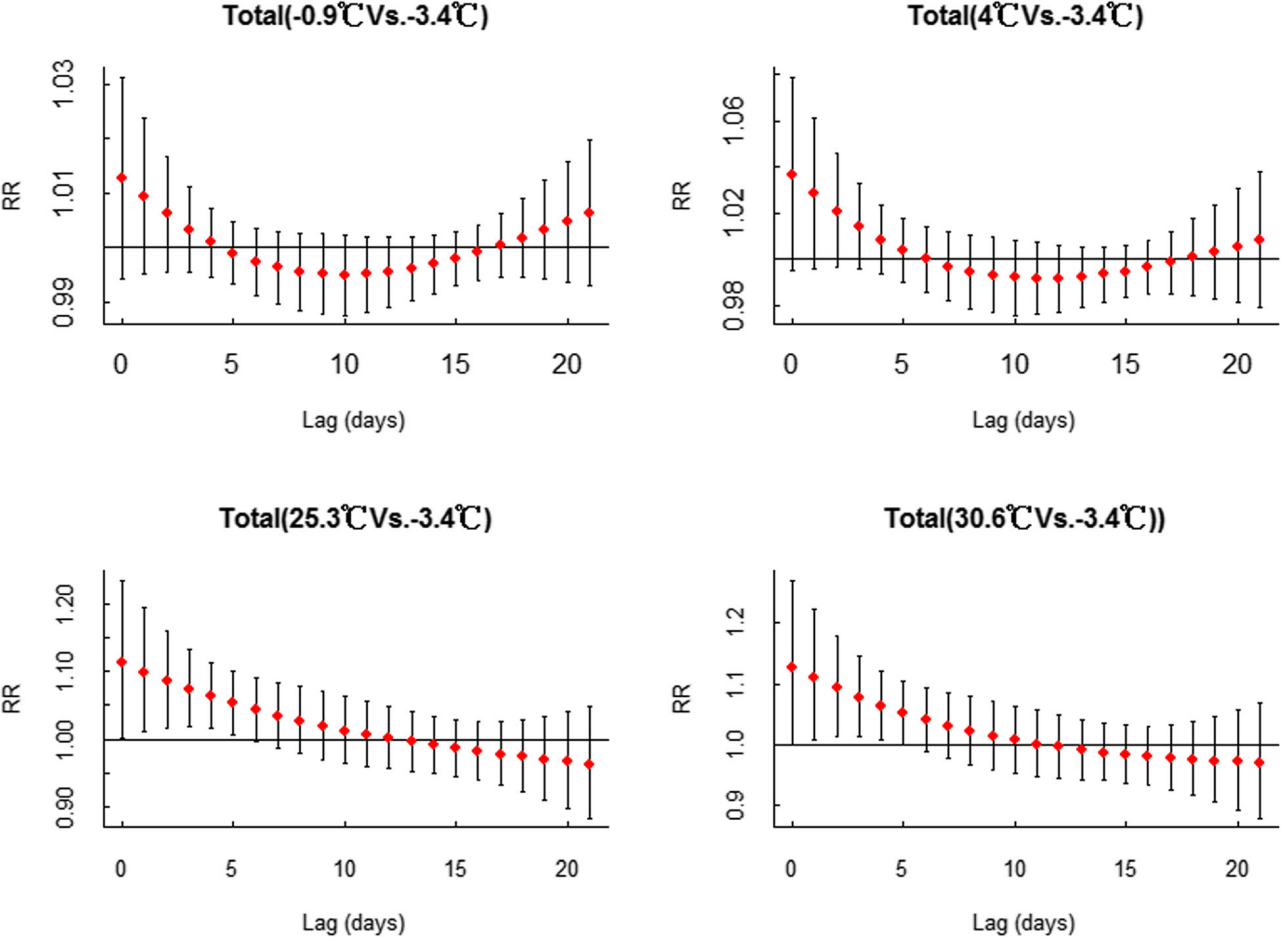
Lag-effects of specific ATs (10th, − 0.9 °C, 25th, 4 °C, 75th, 26.3 °C, 90th, 30.6 °C) on MD emergency admissions, using − 3.4 °C as reference

Dose-response relationships between AT and MDs admissions over different lag days were also examined. The lagged effects from the ATs in different percentiles (P10, − 0.9 °C; P25, 4 °C; P75, 25.3 °C; P90, 30.6 °C) are presented in Fig. 3. With the minimum admission apparent temperature of − 3.4 °C as a reference, the single-day and cumulative lag effects of the 10th and 90th percentiles of AT on MDs admissions are shown in Table 3. In terms of single day lag effects, high AT had the greatest effect (RR = 1.109, 95% CI:1.007–1.222) at lag1, and the effect gradually decreased until the 5th day (RR = 1.051, 95% CI: 1.000–1.105). The cumulative lag effects of high ATs lasted until the 12th day (RR = 1.834, 95% CI: 1.016–3.310). The lagged effects for low AT (both the 10th percentile and the 25th percentile) were not significant. In addition, Additional file 1: Figure S3 shows that specific ATs (P10, − 0.9 °C; P25, 4 °C; P75, 25.3 °C; P90, 30.6 °C) had no significant effect on admissions of patients with MDs due to alcohol.

**Table 3:** Single and cumulative effects estimates at various lag times (in days), with reference of −3.4 °C

Single-day (day(s))	Relative risk (95% confidence interval)		Multi-day (day(s))	Relative risk (95% confidence interval)	
	P10 (−0.9 °C)	P90 (30.6 °C)		P10 (−0.9 °C)	P90 (30.6 °C)
0	1.013 (0.994–1.031)	1.127 (0.999–1.271)	0–0	1.013 (0.994–1.031)	1.127 (0.999–1.271)*
1	1.009 (0.995–1.023)	1.109 (1.007–1.222)*	0–1	1.022 (0.989–1.056)	1.250 (1.006–1.551)*
2	1.006 (0.996–1.017)	1.093 (1.013–1.179)*	0–2	1.028 (0.985–1.073)	1.365 (1.021–1.825)*
3	1.003 (0.995–1.011)	1.077 (1.014–1.145)*	0–3	1.032 (0.981–1.085)	1.471 (1.040–2.079)*
4	1.001 (0.995–1.007)	1.064 (1.009–1.121)*	0–4	1.033 (0.978–1.091)	1.564 (1.062–2.305)*
5	0.999 (0.993–1.005)	1.051 (1.000–1.105)*	0–5	1.032 (0.974–1.093)	1.644 (1.081–2.052)*
6	0.997 (0.991–1.004)	1.040 (0.989–1.094)	0–6	1.029 (0.970–1.091)	1.710 (1.095–2.671)*
7	0.996 (0.990–1.003)	1.030 (0.977–1.086)	0–7	1.025 (0.966–1.088)	1.762 (1.103–2.815)*
8	0.996 (0.988–1.003)	1.022 (0.967–1.079)	0–8	1.020 (0.961–1.084)	1.801 (1.102–2.941)*
9	0.995 (0.988–1.002)	1.014 (0.959–1.072)	0–9	1.016 (0.955–1.080)	1.826 (1.092–3.052)*
10	0.995 (0.988–1.002)	1.007 (0.953–1.065)	0–10	1.010 (0.950–1.075)	1.839 (1.074–3.150)*
11	0.995 (0.988–1.002)	1.001 (0.949–1.057)	0–11	1.005 (0.943–1.071)	1.841 (1.048–3.236)*
12	0.995 (0.989–1.002)	0.996 (0.946–1.049)	0–12	1.001 (0.938–1.068)	1.834 (1.016–3.310)*
13	0.996 (0.990–1.002)	0.992 (0.944–1.042)	0–13	0.997 (0.932–1.066)	1.819 (0.981–3.371)
14	0.997 (0.992–1.002)	0.988 (0.941–1.036)	0–14	0.994 (0.928–1.064)	1.796 (0.944–3.419)
15	0.998 (0.993–1.003)	0.984 (0.938–1.032)	0–15	0.992 (0.925–1.063)	1.768 (0.905–3.452)
16	0.999 (0.994–1.004)	0.981 (0.933–1.032)	0–16	0.991 (0.923–1.064)	1.734 (0.866–3.474)
17	1.000 (0.994–1.006)	0.979 (0.926–1.034)	0–17	0.991 (0.923–1.065)	1.697 (0.826–3.487)
18	1.002 (0.994–1.009)	0.976 (0.917–1.039)	0–18	0.993 (0.924–1.068)	1.657 (0.785–3.498)
19	1.003 (0.994–1.012)	0.974 (0.906–1.047)	0–19	0.996 (0.925–1.073)	1.614 (0.741–3.514)
20	1.005 (9.994–1.016)	0.972 (0.893–1.057)	0–20	1.001 (0.927–1.081)	1.568 (0.694–3.544)
21	1.006 (0.993–1.020)	0.970 (0.881–1.069)	0–21	1.007 (0.929–1.092)	1.521 (0.643–3.598)

*:*P* < 0.05

The lag effects of air pollutants (NO_2_, PM_2.5_, O_3_ and SO_2_) on MDs are demonstrated in Additional file 1: Table S1. A 10-μg/m3 increase, only O_3_ concentrations have an acute effect on MDs on the current day, with the RR of 1.007 (95% CI, 1.001–1.014). However, after adjustments were made for daily mean temperature, this effect became insignificant, with the RR of 1.003 (95% CI, 0.996–1.009).

### Subgroup analysis

**Fig. 4:**
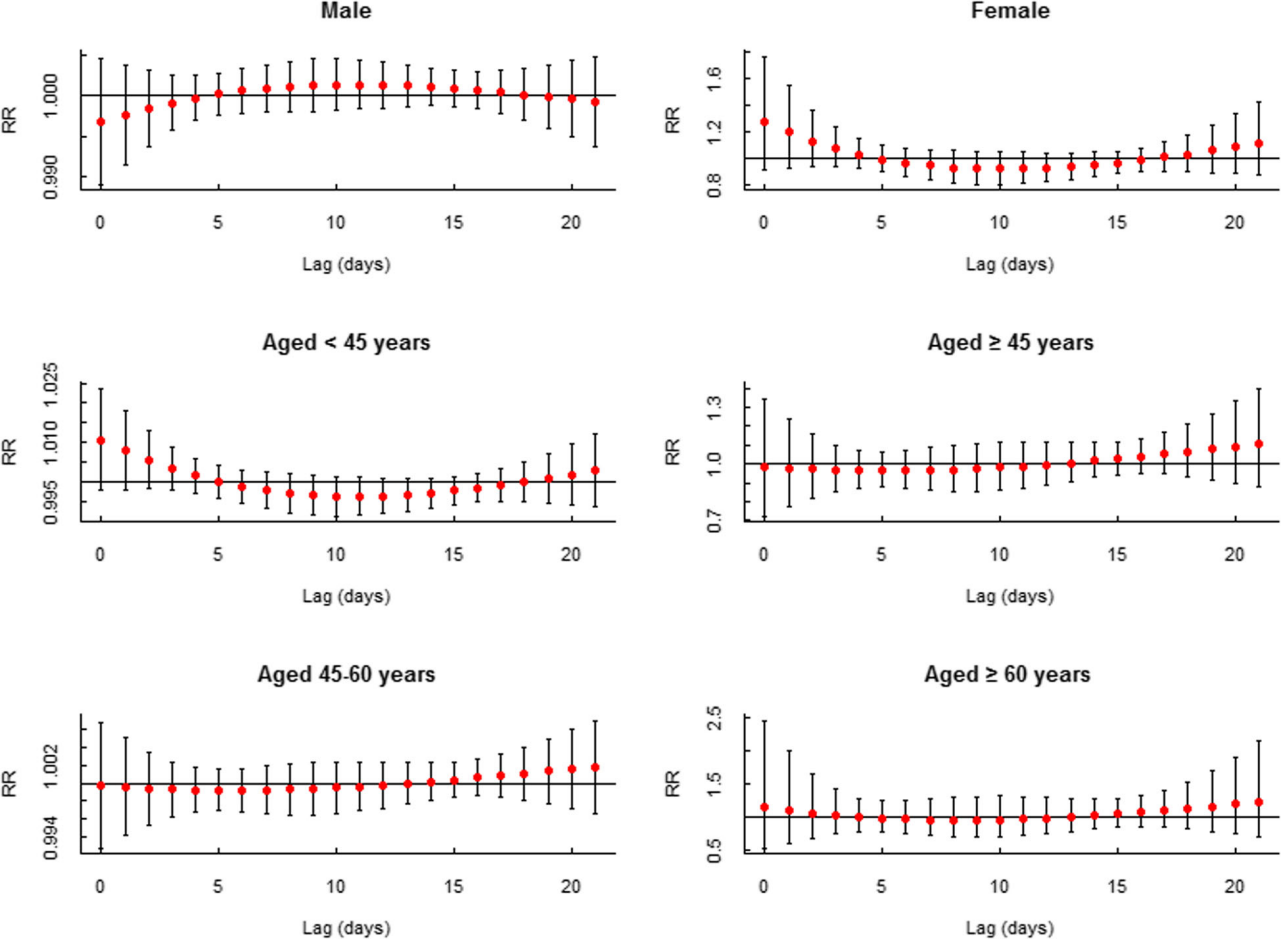
Lag-specific effects of low AT (10th, − 0.9 °C), on mental and behavioral disorders in various subgroups with reference of − 3.4 °C

**Fig. 5:**
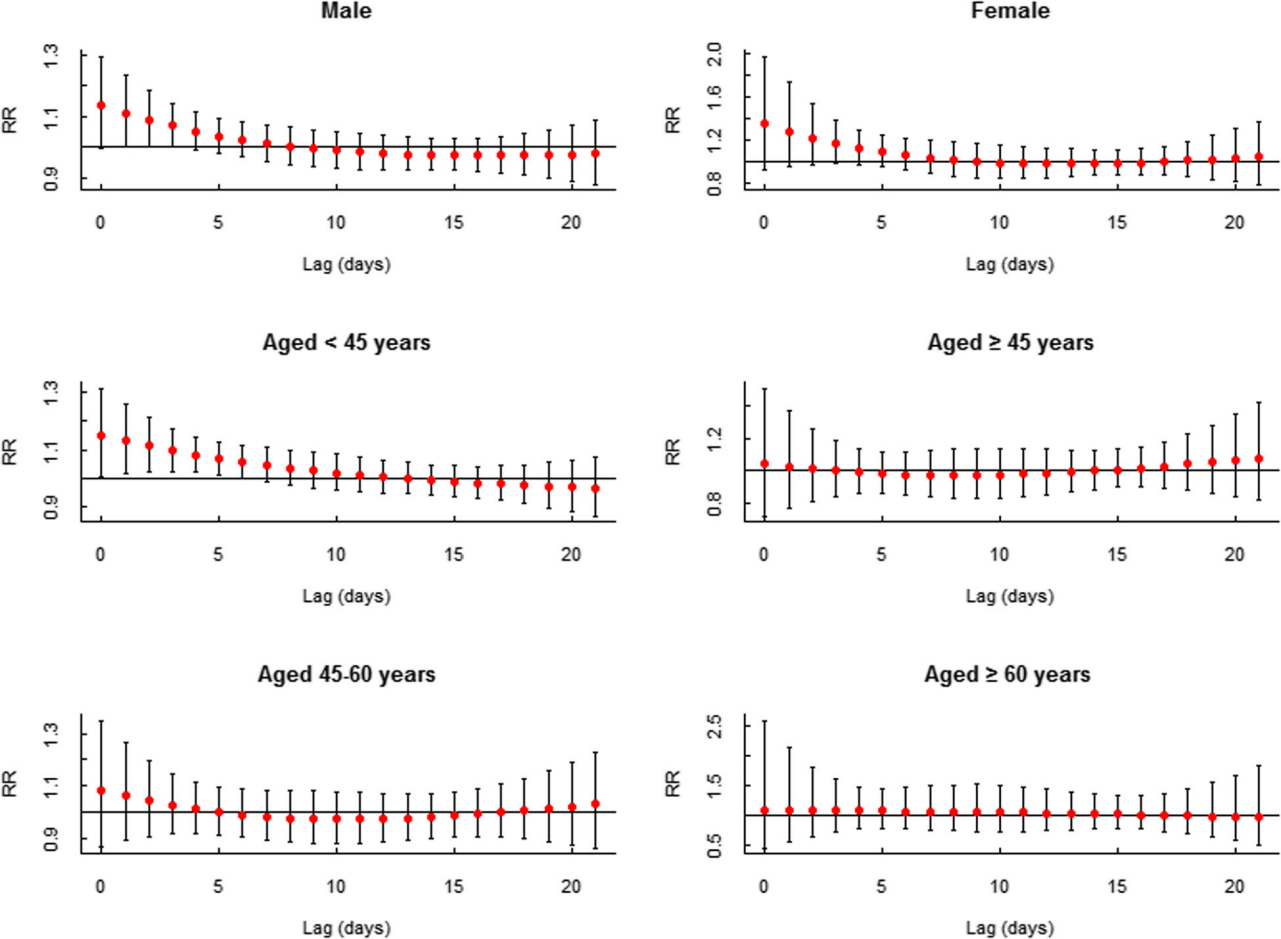
Lag-specific effects of high AT (90th, 30.6 °C), on mental and behavioral disorders in various subgroups with reference of − 3.4 °C

The effects of low AT (P10: − 0.9 °C) on MDs admissions of different subgroups were not statistically significant (Fig. 4). In terms of high AT (P90: 30.6 °C), its estimated effects on males and females were similar, but the 95% confidence interval of female patients was so wide that the effect was insignificant. For different age groups, high AT only showed a significant effect on the group aged < 45 years (Fig. 5).

### Sensitivity analysis

**Fig. 6:**
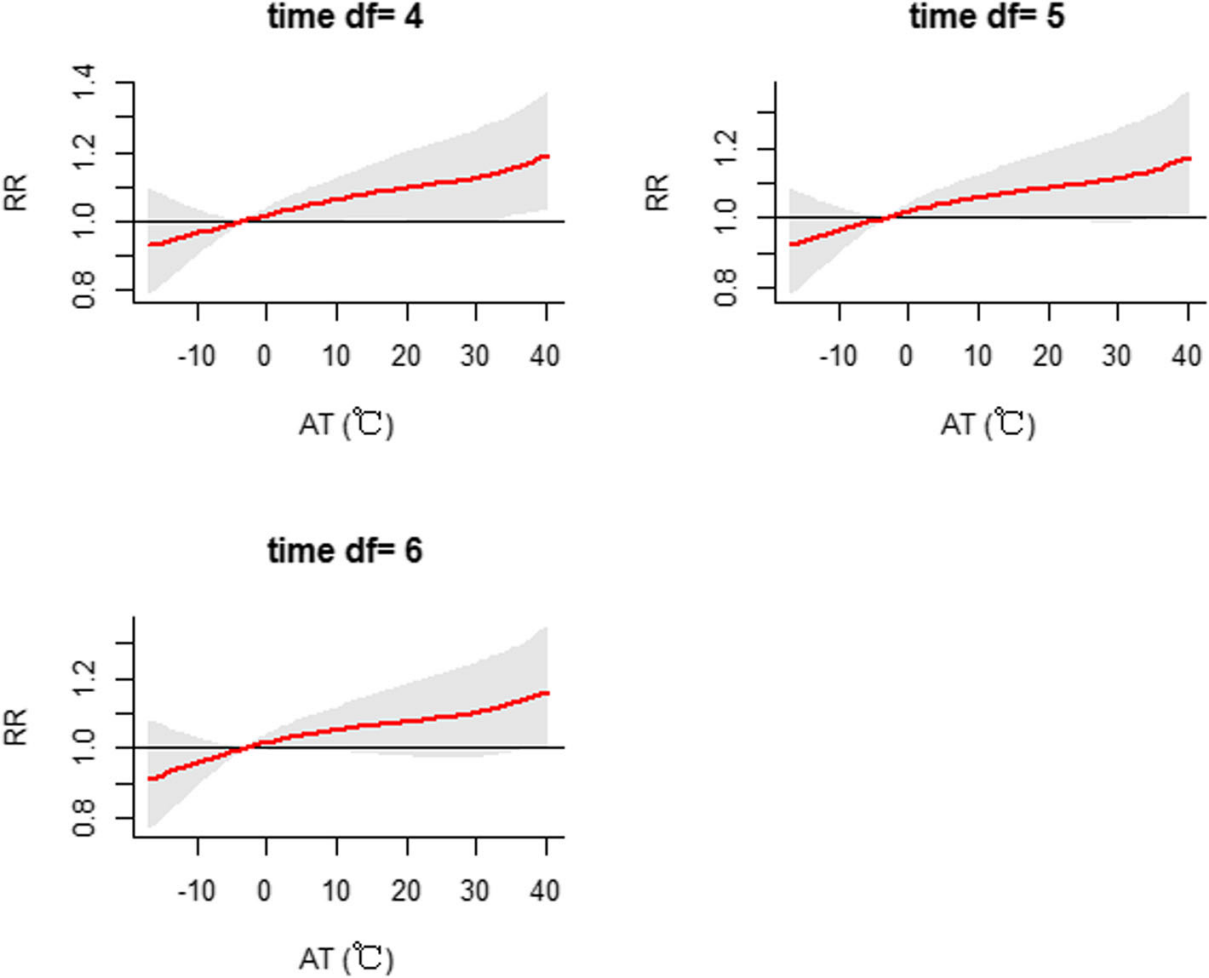
Sensitivity analysis when altering the degrees of freedom (df = 4–6) for controlling for the long-term trend and seasonality in the model

**Fig. 7:**
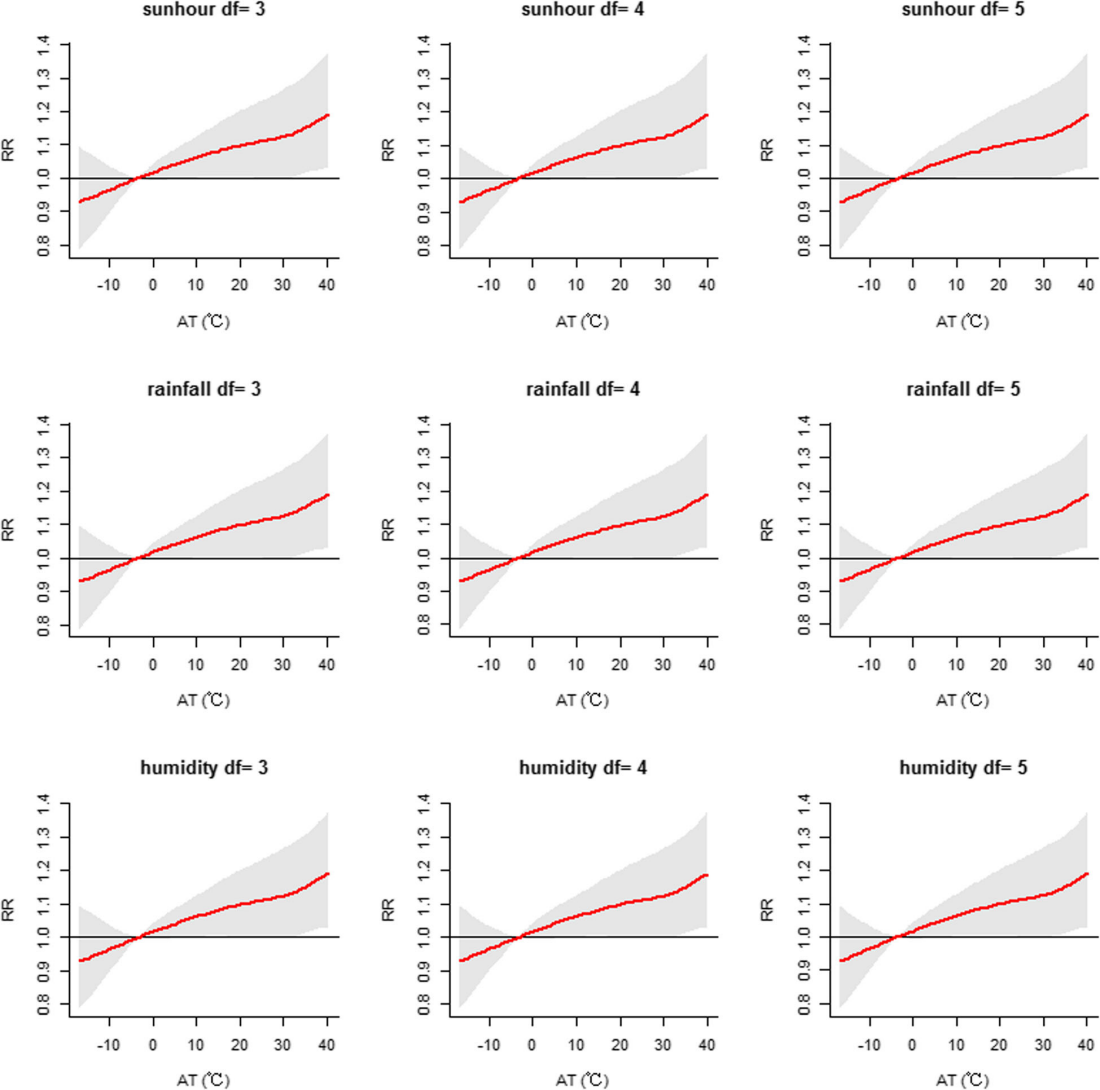
Sensitivity analysis when altering the degrees of freedom (df = 3–5) for rainfall,humidity and sunshine duration in the model Fig. 8Sensitivity analysis when altering the degrees of freedom (df = 3–5) for air pollutants of PM_2.5_ and NO_2_ in the model

**Fig. 8:**
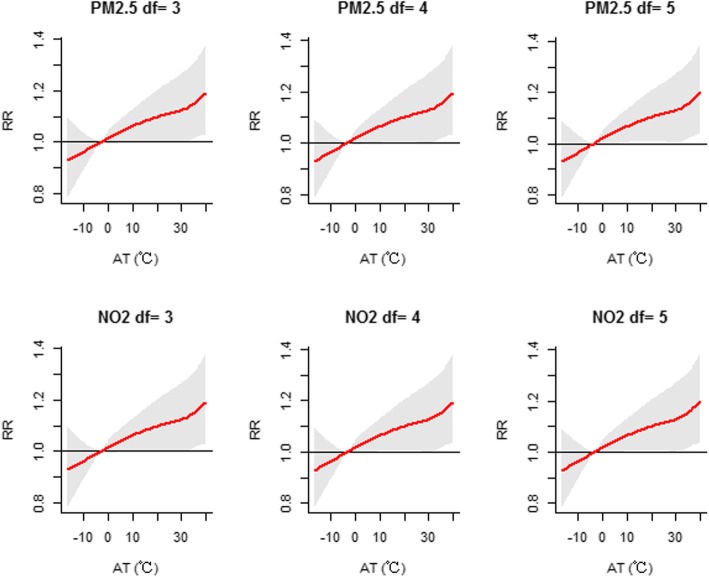
Sensitivity analysis when altering the degrees of freedom (df = 3-5) for air pollutants of PM2.5 and NO2 in the model

**Fig. 9:**
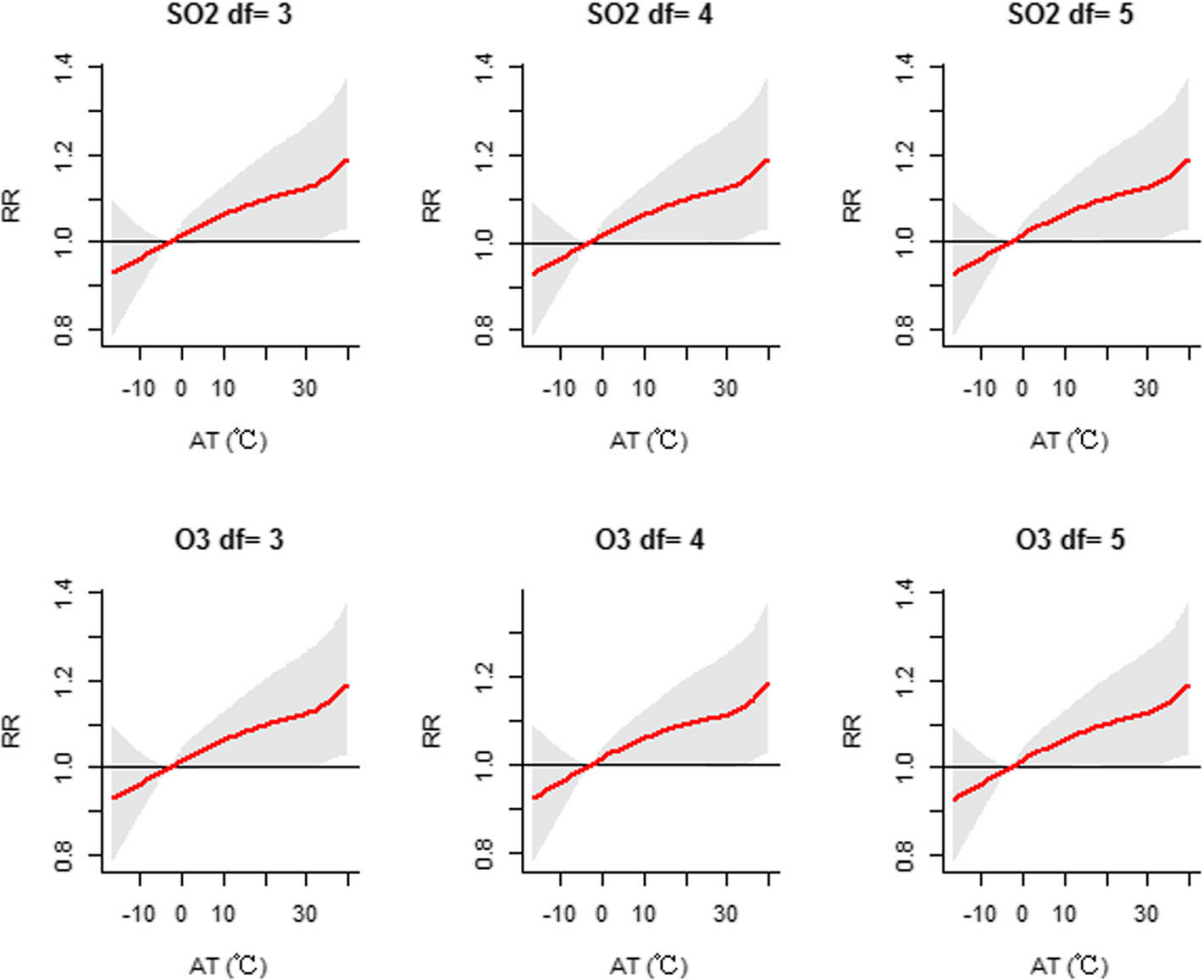
Sensitivity analysis when altering the degrees of freedom (df = 3–5) for air pollutants of SO_2_ and O_3_ in the model

Sensitivity analysis showed that the effects were generally similar when the degrees of freedom were altered for the time trend (df = 4–6), humidity (df = 3–5), sunshine duration (df = 3–5) and air pollutants (df = 3–5) in the model (Figs. 6, 7, 8 and 9). The dose-response curve was similar before and after adjusting for air pollutants (PM_2.5_, SO_2_, NO_2_ and O_3_) (Additional file 1: Figure S4). In view of all of the above results, our analysis was robust. Moreover, we replaced AT with daily mean temperature to compare the two indicators. Additional file 1: Figure S5 demonstrates that the effects of AT were similar to those of the daily mean temperature. Additionally, the values of the Mean Square Error (MSE) and AIC in model 1 when AT was adopted were approximately equal to those in model 2 (adopting daily mean temperature) (Additional file 1: Table S2).

## Discussion

To our knowledge, this study was the first to explore both the non-linear and lagged effects of AT on total MDs emergency hospital admissions in China. We found that high AT had significantly nonlinear and delayed effects on MDs hospital emergency admissions. The effects appeared on the second day and can last for up to 5 days. Moreover, the associations between AT and MDs admissions varied by age and gender, with male patients and patients aged < 45 years being more vulnerable to higher AT (above the 75th percentile). However, no significant association for low AT was observed. Several studies have examined the effects of AT on mental health outcomes. One study in the USA reported that increasing AT was associated with emergency room visits for mental health-related outcomes, except for black and Asian patients [17]. Another study found that both high and low ATs were associated with admissions for schizophrenia in Hefei, China [16]. In addition, several studies have examined the relationship between ambient mean temperature and MDs admissions in Toronto, Canada [9]; Shanghai, China [11]; and Sweden [18]. The higher AT effect observed in our study was consistent with the effect of high ambient mean temperature reported in previous studies but differed from the results by Basu et al. [17]. Reasons for the inconsistency remained unclear, probably because we did not consider MD-related socio-economic factors in our statistical analysis due to data availability.

The potential biological explanations for the association between AT and MDs emergency admission risk varied for different mental disorders. AT is an index combining the environmental temperature, humidity and wind speed. High AT is usually uncomfortable for people, especially for people with mental disorders [19]. In a high-AT (usually with high temperature and low humidity) environment, people with mental disorders may be agitated and become more aggressive and violent, which increases the risk of suicide and conflicts [20–22]. Hotter environments could disturb the metabolites of certain neurotransmitters, such as 5-hydroxytryptamine and dopamine, which are associated with the onset of depression and bipolar disorder [23–25]. In addition, high AT usually represents high environmental temperature, which could indirectly increase the risk of MDs emergency admission by influencing the effect of psychiatric medications used by individuals with mental illness [26]. Furthermore, some psychotropic drugs have side effects related to heat and increase patients’ vulnerability in hot environments [27, 28].

Our paper has several strengths. First, our findings suggest that a combination of meteorological variables, such as ambient temperature, relative humidity and wind speed, significantly affect human mental health and behaviours. The health department, the health surveillance system and mental health institutions should comprehensively take a variety of meteorological factors into consideration when taking measures to decrease the risk of MDs admissions. Second, we included several air pollutants in the analyses that provided strong evidence for health authority. Third, we identified vulnerable MDs patients in this study, providing a specific target population for MDs control and prevention. At the community level, relevant agencies should inform the public of pending high AT weather and how long it could last. Accordingly, they should provide specific advice and open public cooling centres in a timely manner. At the hospital level, due to the lag effects of high AT on MDs emergency admissions, mental health institutions should arrange for emergency preparation in situations of high AT. At the individual level, MDs patients and their families should take note of early warning information, reduce outdoor activities during high AT periods, and turn on air conditioning.

Several limitations also need to be acknowledged. First, our study area was limited to a single city. Therefore, these findings should be interpreted with caution when generalized to other locations. Second, meteorological and pollution data obtained from fixed monitoring stations are approximate estimates of individual exposures, which may lead to an underestimate of correlation. Third, data on specific mental and behavioural disorders, such as depression, was so scarce that we could not perform subgroup analyses for specific mental and behavioural disorders. Moreover, we checked medical records to obtain data; thus, information on some crucial factors, such as socio-economic status and comorbidities, was not available. Finally, this time series analysis was an ecological study, and thus, an ecological fallacy may exist.

## Conclusions

Our study showed that short-term exposure to high apparent temperature was associated with increased MDs emergency admissions in Yancheng, China. More studies are warranted to examine the association between apparent temperature and hospital admissions of patients with mental and behavioural disorders in various regions. In the future, AT might be used as an early warning indicator of emergency admissions of patients with mental and behavioural disorders.

## Supplementary information

**Additional file 1: Table S1.** Results of the lag effects of air pollutants (NO_2_, PM_2.5_, O_3_ and SO_2_) on MDs. **Table S2.** Fitting effects of two models were compared using AIC and MSE. **Figure S1.** Spearman’s correlations between the different meteorological factors and air pollution. **Figure S2.** Time-series distribution of total MDs cases, mean temperature and AT in Yancheng, China, 2014–2017. **Figure S3.** Lag-effects of specific ATs (10th, − 0.9 °C, 25th, 4 °C, 75th, 26.3 °C, 90th, 30.6 °C) on admissions of MDs due to alcohol, using − 3.4 °C as reference. **Figure S4.** Sensitivity analysis before and after air pollutants (PM_2.5_, SO_2_, NO_2_ and O_3_) taken into DLNM model in 2014–2017. (A, before air pollutants were included; B, after air pollutants were included). **Figure S5.** The dose-response relationship of DLNM model, included with AT and daily mean temperature as independent variables, respectively.

## Data Availability

The datasets used and/or analysed during the current study are available from the corresponding author upon reasonable request.

## Abbreviations

AT: Apparent temperature
CI: Confidence interval
DALYs: Disability Adjusted of Life Years
df: degrees of freedom
DLNM: Distributed lag non-linear model
GLM: Generalized linear model
MDs: Mental and behavioral disorders
MSE: Mean Square Error
RR: Relative risk
VIFs: Variance inflation factors
