# Supplementary material for: Effect of apparent temperature on daily emergency admissions for mental and behavioral disorders in Yancheng, China: a time-series study
Source: Environ Health. 2019 Nov 20;18:98. doi: 10.1186/s12940-019-0543-x (PMC6880413; doi:10.1186/s12940-019-0543-x)
Supplement: Supplementary file 1 — Additional file 1: Table S1. Results of the lag effects of air pollutants (NO2, PM2.5, O3 and SO2) on MDs. Table S2. Fitting effects of two models were compared using AIC and MSE. Figure S1. Spearman’s correlations between the different meteorological factors and air pollution. Figure S2. Time-series distribution of total MDs cases, mean temperature and AT in Yancheng, China, 2014–2017. Figure S3. Lag-effects of specific ATs (10th, − 0.9 °C, 25th, 4 °C, 75th, 26.3 °C, 90th, 30.6 °C) on admissions of MDs due to alcohol, using − 3.4 °C as reference. Figure S4. Sensitivity analysis before and after air pollutants (PM2.5, SO2, NO2 and O3) taken into DLNM model in 2014–2017. (A, before air pollutants were included; B, after air pollutants were included). Figure S5. The dose-response relationship of DLNM model, included with AT and daily mean temperature as independent variables, respectively. [file 12940_2019_543_MOESM1_ESM.docx]

Table S1 Results of the lag effects of air pollutants (NO_2_, PM_2.5_, O_3_ and SO_2_) on MDs.

| Lag days | NO_2_ | PM_2.5_ | O_3_ | SO_2_ |
| --- | --- | --- | --- | --- |
|  | RR (95% CI) | RR (95% CI) | RR (95% CI) | RR (95% CI) |
| Lag0 | 1.002(0.986-1.020) | 0.998(0.992-1.004) | 1.007(1.001-1.014)* | 0.983(0.960-1.007) |
| Lag1 | 0.997(0.988-1.006) | 0.999(0.995-1.002) | 1.003(1.000-1.006) | 0.990(0.978-1.002) |
| Lag2 | 0.993(0.985-1.002) | 1.000(0.996-1.003) | 1.000(0.997-1.003) | 0.995(0.984-1.007) |
| Lag3 | 0.992(0.982-1.002) | 1.000(0.997-1.004) | 0.998(0.995-1.002) | 0.998(0.985-1.011) |
| Lag4 | 0.992(0.984-1.001) | 1.000(0.997-1.004) | 0.998(0.995-1.001) | 0.998(0.986-1.010) |
| Lag5 | 0.994(0.987-1.002) | 1.001(0.998-1.004) | 0.998(0.996-1.001) | 0.997(0.988-1.007) |
| Lag6 | 0.997(0.988-1.006) | 1.001(0.997-1.004) | 0.999(0.996-1.002) | 0.995(0.983-1.007) |
| Lag7 | 1.000(0.986-1.016) | 1.001(0.996-1.006) | 1.000(0.995-1.006) | 0.992(0.972-1.013) |

Notes：*P＜0.05

A 10 ug/m3 increase, only O3 concentrations has acute effect on MDs on current day, with effect RR of 1.007 (95% CI: 1.001-1.014). However, after adjusted for daily mean temperature, this effect became insignificant, with effect RRadj of 1.003(95% CI: 0.996-1.009).

Table S2 Fitting effects of two models were compared using AIC and MSE.

|  | Deviance residuals | | | | | Residual deviance | MSE | AIC |
| --- | --- | --- | --- | --- | --- | --- | --- | --- |
|  | Min | *P*25 | Median | *P*75 | Max |  |  |  |
| Model 1 | -3.506 | -0.858 | -0.100 | 0.653 | 3.975 | 1906.0 | 1.376 | 7037.1 |
| Model 2 | -3.536 | -0.841 | -0.095 | 0.659 | 3.976 | 1905.7 | 1.376 | 7036.7 |

Model 1, the DLNM model adopting AT as independent variable; Model 2, the DLNM model adopting

mean temperature as independent variable; MSE, mean squared error; AIC, Akaike Information


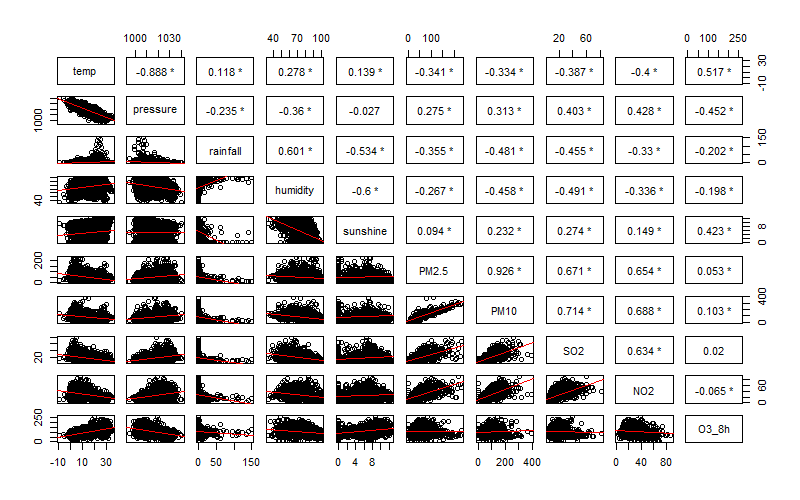


Figure S1. Spearman's correlations between the different meteorological factors and air pollution.

temp: mean temperature; pressure: barometric pressure; sunshine:sunshine duration. *:P < 0.05


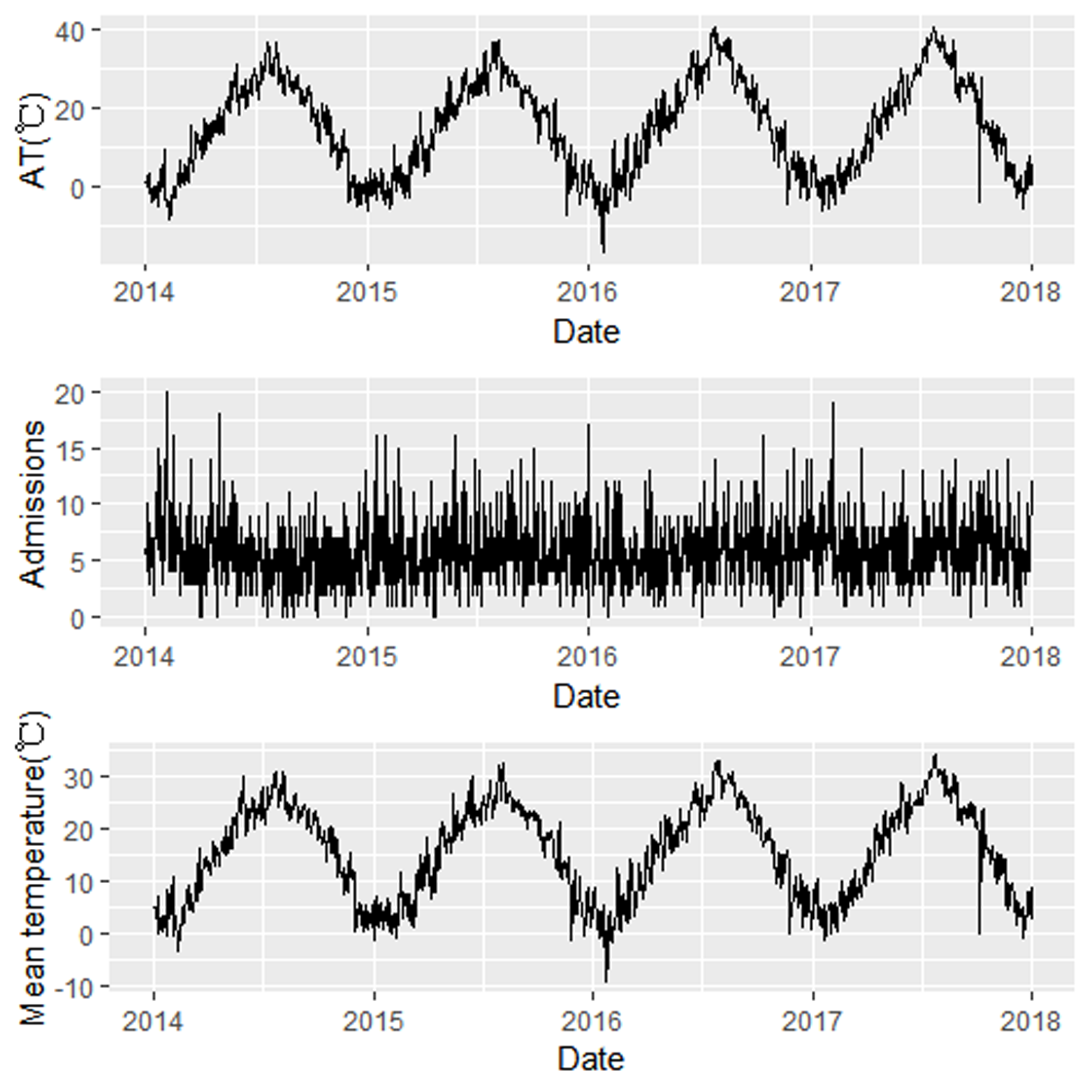


Figure S2. Time-series distribution of total MDs cases, mean temperature and AT in Yancheng, China, 2014-2017.


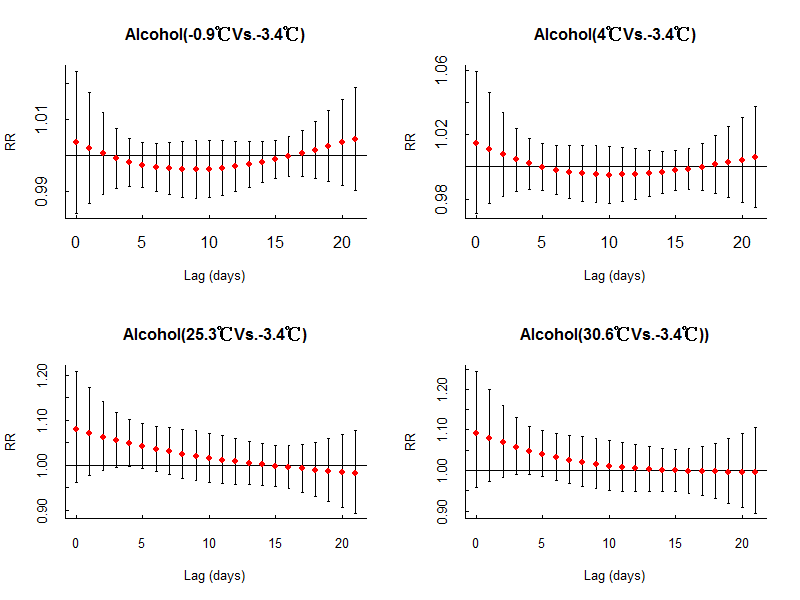


Figure S3. Lag-effects of specific ATs (10th, -0.9℃; 25th, 4℃; 75th, 26.3℃; 90th, 30.6℃) on admissions of MDs due to alcohol, using -3.4℃ as reference.

A


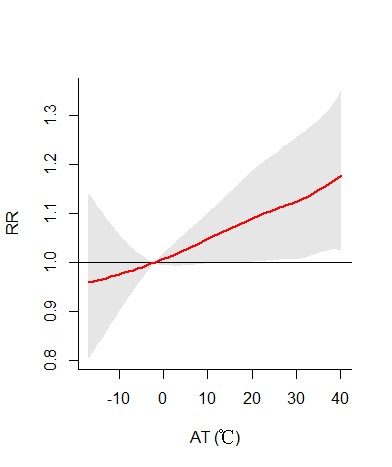


B


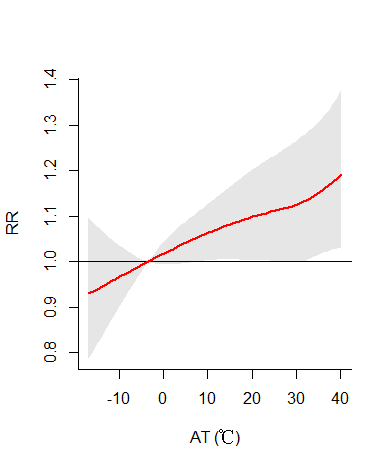


Figure S4.Sensitivity analysis before and after air pollutants (PM_2.5_, SO_2_, NO_2_ and O_3_) taken into DLNM model in 2014-2017. (A, before air pollutants were included; B, after air pollutants were included).


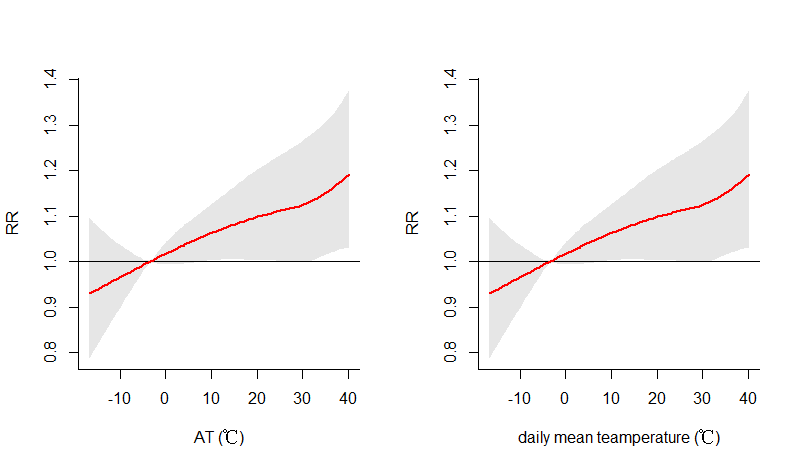


Figure S5. The dose-response relationship of DLNM model, included with AT and daily mean temperature as independent variables, respectively.
